# Spatial Transcriptomics of Nematodes Identifies Sperm Cells as a Source of Genomic Novelty and Rapid Evolution

**DOI:** 10.1093/molbev/msaa207

**Published:** 2020-08-08

**Authors:** Christian Rödelsperger, Annabel Ebbing, Devansh Raj Sharma, Misako Okumura, Ralf J Sommer, Hendrik C Korswagen

**Affiliations:** 1 Department for Integrative Evolutionary Biology, Max Planck Institute for Developmental Biology, Tübingen, Germany; 2 Hubrecht Institute, Royal Netherlands Academy of Arts and Sciences and University Medical Center Utrecht, Utrecht, The Netherlands; 3 Program of Biomedical Science, Graduate School of Integrated Sciences for Life, Hiroshima University, Higashi-Hiroshima, Hiroshima, Japan; 4 Developmental Biology, Department of Biology, Institute of Biodynamics and Biocomplexity, Utrecht University, Utrecht, The Netherlands

**Keywords:** comparative genomics, orphan genes, de novo gene, duplication, *Pristionchus pacificus*, *Caenorhabditis elegans*, meiosis

## Abstract

Divergence of gene function and expression during development can give rise to phenotypic differences at the level of cells, tissues, organs, and ultimately whole organisms. To gain insights into the evolution of gene expression and novel genes at spatial resolution, we compared the spatially resolved transcriptomes of two distantly related nematodes, *Caenorhabditis elegans* and *Pristionchus pacificus*, that diverged 60–90 Ma. The spatial transcriptomes of adult worms show little evidence for strong conservation at the level of single genes. Instead, regional expression is largely driven by recent duplication and emergence of novel genes. Estimation of gene ages across anatomical structures revealed an enrichment of novel genes in sperm-related regions. This provides first evidence in nematodes for the “out of testis” hypothesis that has been previously postulated based on studies in *Drosophila* and mammals. “Out of testis” genes represent a mix of products of pervasive transcription as well as fast evolving members of ancient gene families. Strikingly, numerous novel genes have known functions during meiosis in *Caenorhabditis elegans* indicating that even universal processes such as meiosis may be targets of rapid evolution. Our study highlights the importance of novel genes in generating phenotypic diversity and explicitly characterizes gene origination in sperm-related regions. Furthermore, it proposes new functions for previously uncharacterized genes and establishes the spatial transcriptome of *Pristionchus pacificus* as a catalog for future studies on the evolution of gene expression and function.

## Introduction

The immense morphological diversity across all living organisms has raised the fundamental question, how novelty arises in the first place. Genetic screens in multiple model systems have dissected the underlying architecture of many traits and have highlighted the role of *cis*-regulatory evolution, as well as the emergence of novel genes in shaping phenotypic diversity (Wray 2007; Khalturin et al. 2009). Novel genes can arise from a spectrum of processes ranging from duplication of existing genes to de novo formation from noncoding sequences (McLysaght and Hurst 2016; Rödelsperger, Prabh, et al. 2019; Van Oss and Carvunis 2019). In addition, sequence divergence can contribute to the formation of orphan genes without detectable homologs in other lineages. Such orphan genes make up to one-third of all genes in a given genome (Tautz and Domazet-Lošo 2011; Rödelsperger, Prabh, et al. 2019). Their high abundance combined with the paucity of homologs raises the fundamental question, where orphan genes are coming from and what their biological function might be. Although the origin of orphan genes has been extensively studied in yeast (Carvunis et al. 2012; Vakirlis et al. 2018), vertebrates (Toll-Riera et al. 2008; Knowles and McLysaght 2009), and insects (Levine et al. 2006; Wissler et al. 2013), recent studies in plants and other animal phyla such as nematodes have broadened our knowledge of novel gene formation (Prabh et al. 2018; Prabh and Rödelsperger 2019; Zhang et al. 2019). In particular, nematodes with their small genome sizes and species richness are ideal model systems to study the evolution of novel genes by means of phylogenomic approaches relying on deep taxon sampling (Prabh et al. 2018; Rödelsperger 2018; Rödelsperger, Prabh, et al. 2019). Specifically, each of the two nematode genera *Caenorhabditis* and *Pristionchus* has around 40 described species and continuous sampling efforts discover new species almost every year (Rödelsperger et al. 2018; Herrmann et al. 2019; Stevens et al. 2019). The combination of deep taxon sampling with genome sequencing allowed us to characterize the evolutionary dynamics and regulation of novel genes in *Pristionchus* nematodes as well as to elucidate several mechanisms including de novo formation that give rise to orphan genes (Prabh et al. 2018; Werner et al. 2018; Prabh and Rödelsperger 2019). Moreover, the two nematode species *Caenorhabditis elegans* and *Pristionchus pacificus* have the additional advantage to be established model systems with genetic toolkits including transgenes and genome editing that allow for comparative studies of gene function (Witte et al. 2015; Markov et al. 2016; Moreno et al. 2017, 2018).

In this study, we focus on the spatial expression of novel genes, which can provide further insights into their birth processes and enables to generate hypotheses about their functions. The *P. pacificus* genome has around 8,000 orphan genes without homologs in other nematode families (Rödelsperger 2018; Prabh and Rödelsperger 2019). However, only two of these orphan genes have been functionally characterized in unbiased genetic investigations. First, the orphan gene *dauerless* was found to control the entry into the dauer stage, which represents a highly conserved dispersal and long-term survival strategy in nematodes (Mayer et al. 2015). The second orphan gene *self-1* encodes a small peptide responsible for self-recognition in natural populations of *P. pacificus*. Nematodes of the *P. pacificus* lineage developed tooth-like structures that allow them to predate on other nematodes (Bento et al. 2010; Wilecki et al. 2015). Simultaneously, they evolved a self-recognition system to prevent cannibalism. Notably, a change of a single amino acid in the SELF-1 peptide was sufficient for a mutant line to be killed by its parent (Lightfoot et al. 2019). Although these examples indicate that orphan genes can be identified in unbiased genetic studies, developing rationales for large-scale targeting of novel genes remains difficult.

Therefore, in this study, we focus on the spatial expression of novel genes using RNA tomography (tomo-seq), which can provide insight into their birth processes and allows to generate hypotheses about their functions. Previously, the combination of spatial transcriptomics with knockdown experiments in *C. elegans* provided strong evidence that regional expression was indeed highly indicative for function (Ebbing et al. 2018). To acquire information about regionalized gene expression in *P. pacificus* on a genome-wide scale, we performed spatial transcriptomics of young adult hermaphrodites. First, this allows us to investigate in which anatomical structure a particular gene is expressed. This is of particular importance for the *Pristionchus* research community as spatial expression based on reporter lines is only known for a few *P. pacificus* genes (Ragsdale et al. 2013; Kieninger et al. 2016; Sieriebriennikov et al. 2020) and only a single tissue-specific RNA-seq data set is currently available (Lightfoot et al. 2016). Second, this data set can be used for a comparative analysis of gene expression between *P. pacificus* and *C. elegans*, which have been estimated to have diverged 60–90 Ma (Prabh et al. 2018; Werner et al. 2018). Finally, we can compare the distribution and evolutionary dynamics of genes of different age classes including novel genes across different anatomical regions. This analysis revealed a striking enrichment of novel genes in sperm-related regions, providing first evidence for the “out of testis” hypothesis in nematodes (Levine et al. 2006; Kaessmann 2010; Soumillon et al. 2013; Witt et al. 2019). “Out of testis” genes represent a mix of products of pervasive transcription as well as fast evolving members of ancient gene families. Together, the spatially resolved transcriptome of *P. pacificus* extends our understanding of novel gene formation in nematodes and will be an important resource for future studies of gene function.

## Results

### Spatial Transcriptomics Identifies Thousands of Genes with Regional Expression

**Fig. 1.**
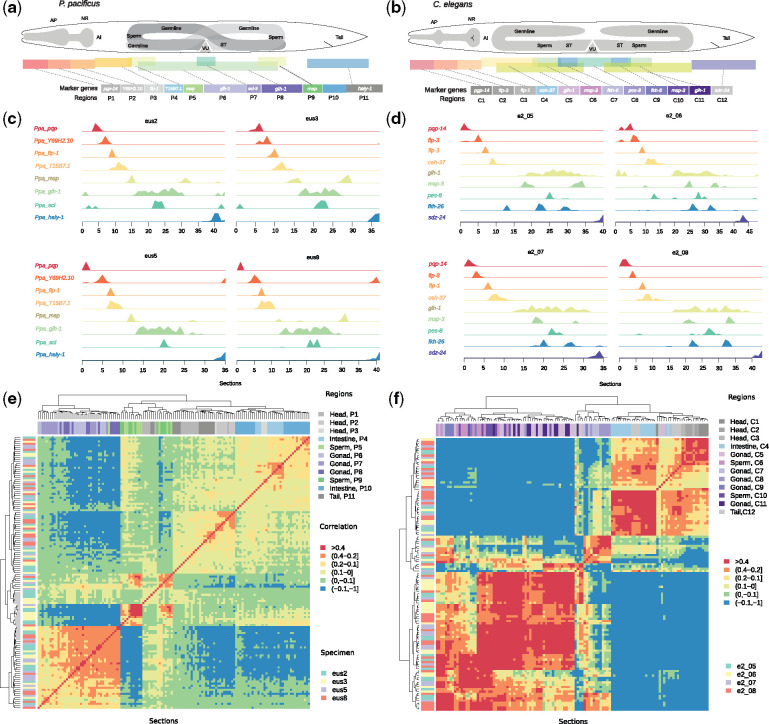
Comparative spatial transcriptomics of nematodes. (*a*, *b*) The schematics represent anatomical overviews of *Pristionchus pacificus* (*a*) and *Caenorhabditis elegans* (*b*) hermaphrodites together with expression domains of selected marker genes which were used to define regions across the A–P axis. For example, P5 was defined as the first peak of *Ppa_msp*, P9 as the second peak, and P10 was defined indirectly as sections between P9 and P11. AP, anterior pharynx region; NR, nerve ring; AI, anterior intestine region; ST, spermatheca; VU, vulva and uterus region. (*c*, *d*) The plots show the normalized expression of marker genes across all sections for four specimens of *P. pacificus* (*c*) and *C. elegans* (*d*). (*e*, *f*) Hierarchical clustering of *P. pacificus* (*e*) and *C. elegans* (*f*) sections based on Pearson correlation indicates that sections within a region are generally highly similar on the whole transcriptome level.

In order to acquire genome-wide gene expression data with spatial resolution, we performed RNA tomography (Junker et al. 2014; Ebbing et al. 2018) which combines cryosectioning of individual *P. pacificus* specimens with RNA sequencing. This approach has been applied previously to compare the spatial transcriptomes of *C. elegans* hermaphrodites and males (Ebbing et al. 2018). In total, four young adult *P. pacificus* hermaphrodites were frozen and cut into around 40 sections per animal (20 µm per section) along the anterior–posterior (A–P) axis. RNA-seq library preparation and sequencing resulted in the detection of 21,046–32,930 (median of all sections per animal) transcripts per section. In total, we detected 12,265 *P. pacificus* genes with expression in all four specimens (supplementary table S1, Supplementary Material online). Reanalysis of the spatial transcriptomics data from *C. elegans* hermaphrodites with the same filtering criteria yielded 6,890 robustly expressed genes (Ebbing et al. 2018). We used orthologs of previously established *C. elegans* marker genes with punctuated spatial expression to define distinct regions (P1–P11 in *P. pacificus* and C1–C12 in *C. elegans*) that would correspond to different anatomical structures (fig. 1*a* and *b*) (Ebbing et al. 2018). For example, the neuropeptide encoding gene *flp-1* (P3, C3), which in *C. elegans* is expressed in the nerve ring (Nelson 1998), defines regions P3, C3, and the anterior regions (P1, P2, C1, and C2) as different parts of the head (fig. 1*a* and *b*). The gonads including the germline are defined by expression of the helicase *glh-1* (P5–P9 and C5–C11) (Gruidl et al. 1996). Within the gonads, the expression of major sperm protein (MSP) genes marks sperm-related regions (P5, P9 and C6, C10) (Ebbing et al. 2018; Tzur et al. 2018). In contrast to males, spermatogenesis in hermaphrodites occurs in a finite duration during development. In *P. pacificus* hermaphrodites, spermatogenesis is delayed relative to *C. elegans* hermaphrodites and starts only at the last larval molt, with the transition to oogenesis occurring 4–6 h into adulthood (Rudel et al. 2005). As mature sperm, which are stored in the spermatheca (fig. 1*a* and *b*), are transcriptionally and translationally silent, the detected MSP expression rather represents residual mRNA levels than de novo transcription. Regions P4 and C4 are enriched for signals from the intestine that are not intermingled with either neuronal or germline expression. The comparison of the spatial transcriptome with previously generated *P. pacificus* reporter lines showed a high level of agreement (supplementary fig. S1, Supplementary Material online) (Ragsdale et al. 2013; Kieninger et al. 2016; Sieriebriennikov et al. 2020). For example, *eud-1*, the master regulator of the mouth form dimorphism in *P. pacificus* (Ragsdale et al. 2013; Kieninger et al. 2016), which is expressed in a few head neurons, is exclusively enriched in region P2 (supplementary fig. S1, Supplementary Material online). Importantly, the expression profiles of many of these marker genes are highly similar across species and individuals (fig. 1*c* and *d*). Taken together, these first analyses indicate that our data set represents a high-quality, spatially resolved transcriptome of *P. pacificus* that is suited for comparative analysis with *C. elegans*.

Based on the partitioning into regions, we summarized expression values per gene by taking the median *z*-score for all sections in a given region. We further defined regional genes based on a relative enrichment of expression in a given region (median *z*-score >1). This identified 3,502 regional genes in *P. pacificus* (supplementary table S3, Supplementary Material online) and 3,656 for the reanalyzed *C. elegans* data (supplementary table S4, Supplementary Material online) (Ebbing et al. 2018). Next, we performed a clustering approach to assess whether regions that reflect similar anatomical structures (as defined using only a few marker genes) show similar transcriptomic profiles on a global scale. Despite the variable degrees of cellular heterogeneity across sections, hierarchical clustering based on Pearson correlation grouped individual sections by anatomical region and not by specimen (fig. 1*e* and *f*). Thus, anatomical regions identified in individual animals are reproducible across different animals, and they are not affected by batch effects or individual-to-individual variation. Together, our data set of 3,502 genes with regional expression profiles represents an enormous extension to the knowledge about spatial expression in *P. pacificus*.

### Regional Expression Is Not Highly Conserved between *P. pacificus* and *C. elegans*

**Fig. 2.**
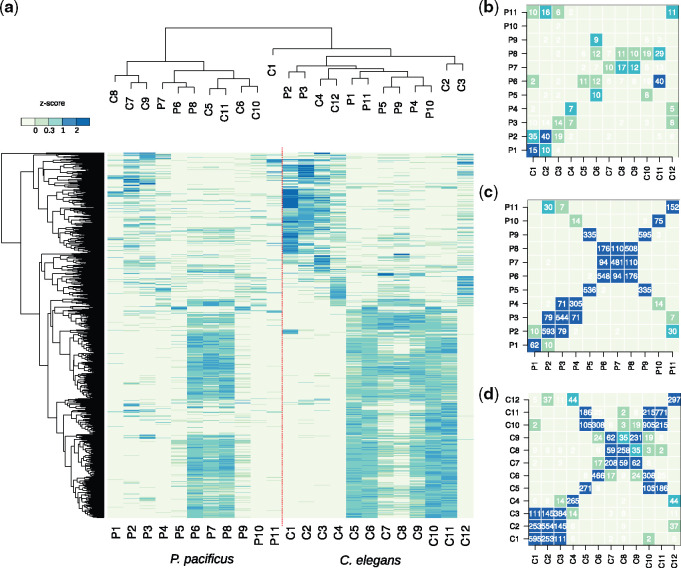
Limited conservation of highly spatially restricted expression. (*a*) The heatmap shows the median *z*-score-normalized expression values for one-to-one orthologs of regional genes in both species. Joint clustering of regions separates the germline from the rest of the worms. (*b*–*d*) The heatmaps show the numbers of shared one-to-one orthologs (*b*) or shared genes in *Pristionchus pacificus* (*c*) and *Caenorhabditis elegans* (*d*) between different regions. The color code scales with the significance of the overlap (Fisher’s exact test).

The availability of spatially resolved and highly comparable transcriptomes of *C. elegans* and *P. pacificus* provides the opportunity to study the evolution of regional expression in nematodes. Despite the high conservation of expression patterns of individual marker genes such as *flp-3*, *glh-1*, and MSP genes and their orthologs in *P. pacificus* (fig. 1*c* and *d*), it remains elusive to what extent regional expression is generally conserved between species. In addition, since partially nonorthologous marker genes were selected to segment the spatial transcriptomes of *P. pacificus* and *C. elegans* (fig. 1*a*–*d*), it is unclear how the different regions of both species correspond to each other. To assess the overall degree of similarity between different regions across species, we compared expression profiles of regional one-to-one orthologs. We combined *z*-score normalized expression data of 1,559 one-to-one orthologous genes with regional expression in at least one of the species and performed a clustering analysis (fig. 2*a*). The most dominant signal is the separation between the gonadal regions (P6–P8, C5–C11) and the rest of the worms. Specifically, the majority of genes is highly expressed in the gonadal regions of both species resulting in a joint clustering of data from both species (fig. 2*a*). In contrast, outside the gonadal regions, the clustering appears to be predominantly driven by species-specific signals, such as varying levels of intestinal or cuticular expression and similarities between head and tail regions (fig. 2*a*). Interestingly, the most anterior regions of *C. elegans* (C1–C4) show hundreds of genes with highly specific expression that are absent in *P. pacificus* suggesting substantial divergence between the two species. These genes are mostly involved in muscle-related functions, such as mitochondrion (*P* < 10^−15^), striated muscle (*P* < 10^−11^), and locomotion (*P* < 10^−3^) (Huang et al. 2009). This difference is consistent with a recent morphological study showing a pronounced shift in the *P. pacificus* lineage from muscular to glandular tissues in the mouth regions (Riebesell and Sommer 2017). To quantify the amounts of regional genes with highly conserved expression more explicitly, we counted the number of shared one-to-one orthologs in every pairwise comparison (fig. 2*b* and supplementary table S5, Supplementary Material online). Surprisingly, only a few dozens of one-to-one orthologs are shared between regions. This finding suggests substantial expression divergence, because overall, 25–30% of the *P. pacificus* genes have one-to-one orthologs in *C. elegans* (Rödelsperger, Athanasouli, et al. 2019). Thus, regionally expressed genes seem to be largely composed of one-to-one orthologs with divergent expression and novel genes.

### Conserved and Symmetric Expression Patterns Support the Homology of Anatomical Structures between *P. pacificus* and *C. elegans*

Although the overall level of conserved regional expression is low, the comparison of regional expression based on shared orthologs also provides some indication how the different regions in both nematodes correspond to each other. In particular, the head and tail regions exhibit a high similarity across the data sets of both species. For example, the *P. pacificus* region P2 shares 40 regional one-to-one orthologs with *C. elegans* region C2, but also 35 with *C. elegans* region C1 (fig. 2*b*). This shows that there is no unambiguous mapping between individual regions in the anterior part of *P. pacificus* to their counterparts in *C. elegans*. However, as a single unit, the three most anterior *P. pacificus* regions together (P1–P3) share dozens of one-to-one orthologs specifically with the most anterior regions in *C. elegans* (C1–C3) and thus, represent distinct segments of the heads (fig. 2*b*). Also, the intestinal regions P4 and C4 from both species seem to correspond to one another, and the most posterior regions (P11 and C12) show highly significant sharing of one-to-one orthologs (fig. 2*b*). Within regions representing the gonads (consisting of both the somatic and germline gonad), the signal is obscured partially by the symmetry of gonad structures and the anatomical differences between the species (fig. 1*a* and *b*). To illustrate this, we tested for gene sharing between regions of the same species. This perfectly recapitulated the symmetry of the gonads, as many of these regions exhibit highly significant gene sharing with themselves and one additional region. For example, both MSP expressing regions (P5 and P9 in *P. pacificus* and C6 and C10 in *C. elegans*) have hundreds of genes in common (fig. 2*c* and *d*). In the case of *C. elegans*, sperm-related signals seem to be stronger mixed with general gonadal expression than in *P. pacificus* because the global transcriptomic profiles in *C. elegans* are more similar to other gonadal regions and the amount of shared regional genes is higher (figs. 1*e*, 1*f*, and 2*d*). Thus, even though the overall level of conserved regional expression is rather low, it is still sufficient to identify homologous anatomical regions across species as follows: head, intestine, general gonad, sperm-related, and tail regions.

### Spatial Expression Patterns Reveal Regional Clustering of Large Gene Families

**Fig. 3.**
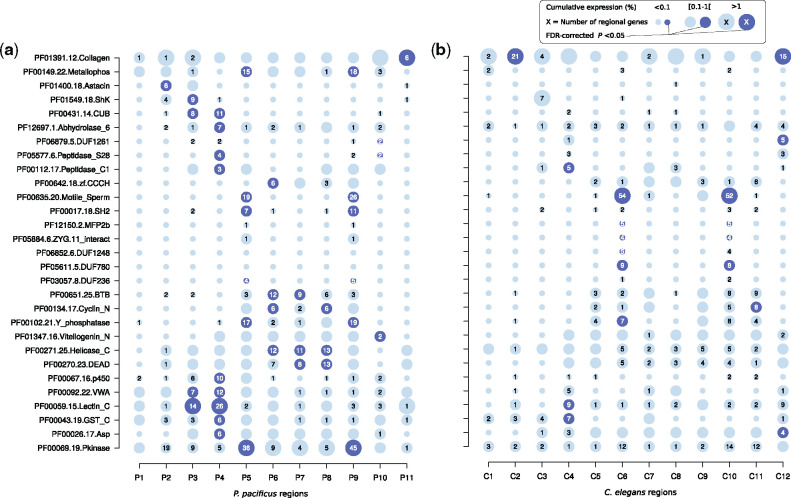
Overrepresentation of protein domains among regional genes. (*a*, *b*) We identified 29 protein domains that are significantly enriched in regional genes in at least one of the species. The plots show the number of regional genes, the cumulative expression, and an indication of significance for a given protein domain in *Pristionchus pacificus* (*a*) and *Caenorhabditis elegans* (*b*).

The limited degree of conservation at the level of one-to-one orthologous genes (fig. 2*b*) is indirect evidence that certain novel gene families are enriched among regional genes. This would be consistent with a previous studies showing that large portions of developmentally regulated genes in *C. elegans* and *P. pacificus* originated from lineage-specific duplication events and therefore, do not have one-to-one orthologs (Castillo-Davis and Hartl 2002; Baskaran et al. 2015). Thus, we tested regional genes for an overrepresentation of gene families as defined by the presence of certain protein domains (fig. 3*a* and *b*). Consistent with previous analysis based on reporter lines, we found a significant enrichment (false discovery rate-corrected *P* value <0.05) of astacin genes in the head region P2 of *P. pacificus* (Sieriebriennikov et al. 2020), which is not observed in the *C. elegans* data (fig. 3*a* and *b*). This example of expression divergence might be an indirect effect caused by cellular heterogeneity across species as the corresponding gland cell expressing these astacins is greatly expanded in *P. pacificus* (Riebesell and Sommer 2017; Sieriebriennikov et al. 2020). Overall, this analysis revealed that most regions are strongly biased toward the expression of members of a particular gene family. The numbers of such lineage-specific duplicates can by far exceed the amount of one-to-one orthologs in a given region (fig. 3*a* and *b*). For example, although regions P4 and C4 only share seven one-to-one orthologs (fig. 2*b*), P4 and C4 contain 26 and 9 regionally expressed C-type lectins. C-type lectins have been implicated in the innate immunity of *C. elegans* and were previously found to be expressed in the intestines of both nematodes (Schulenburg et al. 2008; Lightfoot et al. 2016). Similarly, MSP expressing regions share almost no one-to-one orthologs, yet both nematode species express between 19 and 54 different MSPs in a regional manner (fig. 3*a* and *b*). Thus, gene families that have undergone recent duplications show strong regional clustering in the spatial transcriptomes of *P. pacificus* and *C. elegans*.

### Previously Uncharacterized Gene Families Are Expressed in Sperm-Related Regions

Although many of the enriched gene families such as C-type lectins, MSPs, vitellogenins, and kinases have well-characterized molecular or biological functions, we observed multiple domains of unknown function (DUF) with regional expression as well (fig. 3*a* and *b*). DUFs mostly represent evolutionary constrained sequence stretches in lineage-specific clusters of orthologous and paralogous genes (El-Gebali et al. 2019). On a functional level, these novel gene families are still poorly characterized, probably because different paralogs are functionally redundant or because large-scale RNAi screens did not yield obvious developmental or morphological phenotypes (Kamath et al. 2003). For example, the spatial expression profile of DUF1261-containing genes is suggestive of intestinal expression (fig. 3*a* and *b*) and indeed five of them were previously detected in the intestinal transcriptome of *C. elegans* (Lightfoot et al. 2016). Interestingly, DUF780, DUF236, and DUF1248 show the highest signal in MSP expressing sperm-related regions in at least one of the species (fig. 3*a* and *b*). It is important to note that all these gene families are largely restricted to nematodes and thus, homologous sequences are unknown from vertebrates and most insects (El-Gebali et al. 2019). Following the idea that regional expression could be an indicator of potential biological function (Ebbing et al. 2018), we would hypothesize that the coexpression of the three gene families DUF780, DUF236, and DUF1248 together with MSPs possibly indicates a role in sperm cells (fig. 3*a* and *b*).

### The Phylogenies of Sperm-Related Gene Families Are Consistent with Recent Expansions

**Fig. 4.**
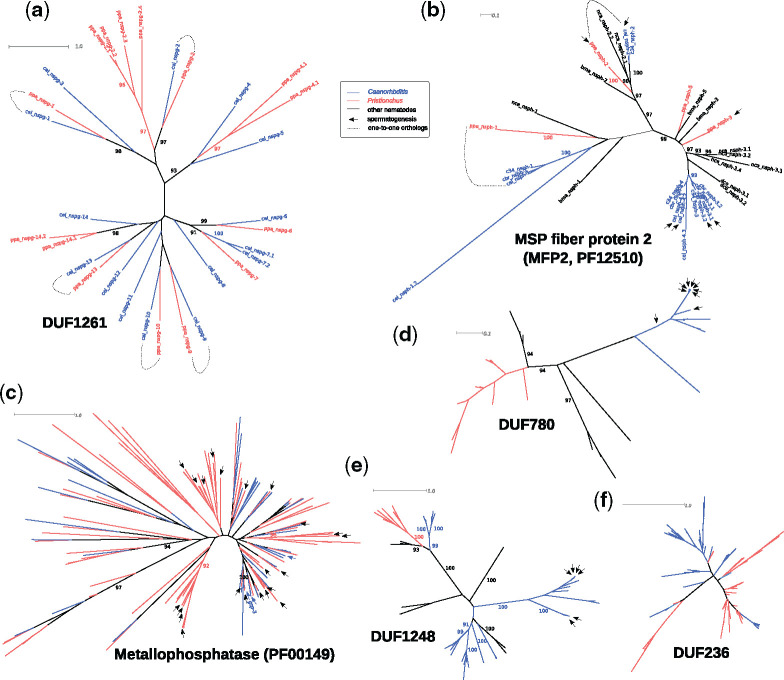
Rapid gene expansion of sperm-related gene families. (*a*) DUF1261 represents an uncharacterized gene family that is expressed in the nematode intestine. A number of genes within this gene family are preserved as one-to-one orthologs between *Pristionchus pacificus* and *Caenorhabditis elegans*. (*b*, *c*) Members of the MFP2 (*b*) and metallophosphatase (*c*) gene families are known to be involved in spermatogenesis and sperm motility. Multiple sperm-related genes in both families are found in lineage-specific clusters and likely arose by recent gene duplication. (*d*–*f*) The phylogenies of other uncharacterized gene families also exhibit strong signatures of recent gene expansions and sperm-related genes are found in *Caenorhabditis*-specific subtrees of DUF780 (*d*) and DUF1248 (*e*). DUF236 containing genes usually have multiple repeated DUF236 domains; the phylogenetic tree (*f*) represents the relationship of individual repeat units. Thus, individual sperm-related genes cannot be indicated.

Given that reproductive genes are frequently associated with rapid evolution (Swanson and Vacquier 2002; Kasimatis and Phillips 2018), we tested whether the phylogenies of the corresponding gene families are indeed consistent with rapid evolution and exhibit signatures of recent lineage-specific gene expansions. To this end, we reconstructed phylogenies of members of all three families and compared these with the phylogeny of DUF1261-containing proteins (as an example for a DUF family without sperm-related expression profile, fig. 4*a*). In addition, we generated phylogenies for two other gene families that are also coexpressed with MSPs, metallophosphatases including *C. elegans gsp-3* and homologs of MSP fiber protein 2 (MFP2), which are involved in sperm motility (Buttery et al. 2003; Wu et al. 2012) (fig. 4*b* and *c*). This analysis revealed that individual members of the DUF1261 gene family are perfectly preserved as one-to-one orthologs between *P. pacificus* and *C. elegans* (fig. 4*a*). In contrast, the two sperm-related gene families are consistent with recent gene expansions (fig. 4*b* and *c*). For example, four out of five of the sperm-related MFP2 genes from *C. elegans* arose very recently from duplication after the split from its sister species *Caenorhabditis inopinata* (C. sp34, fig. 4*b*) (Kanzaki et al. 2018). Similarly, sperm-related metallophoshophatases in *P. pacificus* tend to cluster in lineage-specific groups of paralogs which is a common sign of recent duplications. Also, in the case of the three uncharacterized but putatively sperm-related gene families DUF780, DUF236, and DUF1248, clusters of paralogous genes most-likely point toward recent lineage-specific duplication (fig. 4*d*–*f*). We conclude from these observations that the phylogenies of all five sperm-related gene families show signatures of recent expansions that are consistent with rapid evolution.

### Spatially Resolved Phylostratigraphy Supports the *Out of Testis* Hypothesis in Nematodes

**Fig. 5.**
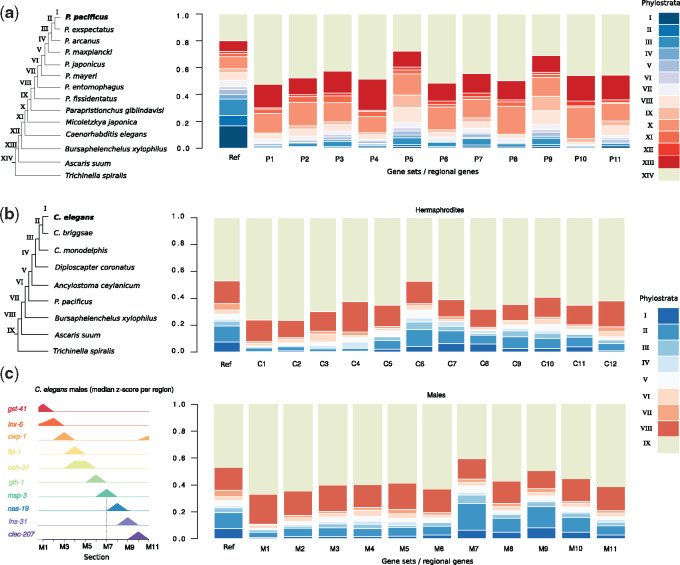
Expression of novel genes in sperm-related regions. (*a*, *b*) We classified *Pristionchus pacificus* genes into gene ages by means of phylostratigraphy and plotted the fraction of phylostrata across the different regional gene sets. The stacked bars labeled as “Ref” show the genome-wide, phylostratigraphic distribution which is dependent on the phylogenetic resolution around the focal species. Sperm-related regions in *P. pacificus* (*a*, P5, P9) and *Caenorhabditis elegans* (*b*, C6, C10) showed the highest fraction of young age classes which is consistent with the “out of testis” hypothesis. (*c*) Reanalysis of tomo-seq data of *C. elegans* males identifies 11 regions (M1–M11) and confirms that the MSP expressing region (M7) indeed shows the highest fraction of young phylostrata.

Genomic studies in *Drosophila* and mammals revealed that novel genes frequently exhibit testis-biased expression, resulting in the “out of testis” hypothesis, which postulates that testis might represent a special environment facilitating the formation of novel genes (Levine et al. 2006; Soumillon et al. 2013; Witt et al. 2019). The above-mentioned examples of the novel gene families (DUF780, DUF236, and DUF1248) that are expressed in sperm-related regions provide first evidence that the “out of testis” hypothesis may also hold true in nematodes. To provide additional support for the “out of testis” hypothesis in nematodes, we systematically assessed the amount of novel genes that are expressed in different anatomical structures, by performing a phylostratigraphic analysis of all regionally expressed genes (Domazet-Loso et al. 2007). To this end, we combined comparative genomic data from multiple non-*Pristionchus* species with the genomes of seven additional *Pristionchus* species that were recently sequenced on the same platform with similar protocols to study the evolutionary dynamics of novel gene families (Prabh et al. 2018) (fig. 5*a*). The underlying ladder-like phylogeny enables us to date the emergence of a gene based on the presence/absence patterns of homologous sequences in other taxa. This defined phylostrata (age classes), which can be quantified across anatomical regions (fig. 5*a*). For example, phylostratum I denotes genes that are specific to *P. pacificus*, whereas genes of other phylostrata are shared with more distantly related species and are therefore to be considered older. The *P. pacificus* data set supports the “out of testis” hypothesis as regions P5 and P9 show the overall highest ratio of young phylostrata (I–XIII, fig. 5*a*). We repeated the phylostratigraphic analysis for *C. elegans* using a different phylogenomic context, which included the high-quality *Caenorhabditis briggsae* genome and the most basal *Caenorhabditis* species *C. monodelphis* (Stein et al. 2003; Slos et al. 2017). Also, in this data set, the sperm-related regions (C6 and C10) exhibit the highest fraction of young phylostrata (I–VIII, fig. 5*b*). To further support this observation, we reanalyzed tomo-seq data of *C. elegans* males (Ebbing et al. 2018). Consistent with the fact that *C. elegans* males have only one gonadal arm, we observe a single peak of young phylostrata in region M7 which also expresses MSP genes (fig. 5*c*). Interestingly, region M9 that overlaps the seminal vesicle and vas deferens (Ebbing et al. 2018) also exhibits high levels of young phylostrata suggesting a role of novel genes in sperm maturation and seminal fluid function (fig. 5*c*). To further narrow down the location of highest expression of novel genes, we repeated this analysis with a previously published *C. elegans* tomo-seq data set consisting of ten gonadal regions (Tzur et al. 2018). Although expression of MSP was not detectable in hermaphrodite data of the investigated developmental stage (L4 + 24 h), we observed a pronounced peak of young phylostrata in regions of crossover formation and desynapsis (supplementary fig. S2, Supplementary Material online). These findings indicate that meiotic spermatocytes exhibit the highest levels of young phylostrata and they are consistent across data sets generated from different laboratories, nematode sexes, and species. Together, our observations provide strong support for the “out of testis” hypothesis in nematodes.

### Young *Out of Testis* Genes May Result from Pervasive Transcription and Evolve Rapidly

**Fig. 6.**
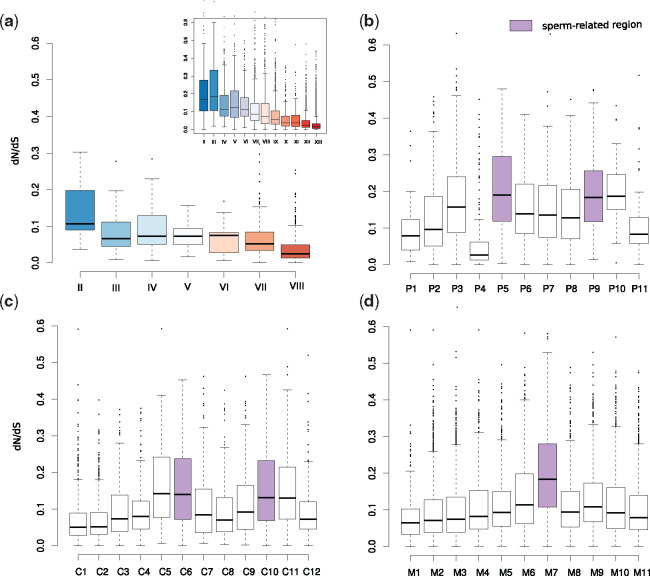
Relaxed evolutionary constraint in sperm-related regions. (*a*) The boxplots show median and interquartile ranges of d*N*/d*S* ratios inferred from orthologs between *Caenorhabditis elegans* and *C. briggsae* for different phylostrata. The inset shows equivalent data for *Pristionchus pacificus* and *P. exspectatus* orthologs. Generally, older genes underlie stronger purifying selection than more recent gene classes. (*b*–*d*) The distribution of d*N*/d*S* values across sets of regional genes in *P. pacificus* hermaphrodites (*b*), *C. elegans* hermaphrodites (*c*), and *C. elegans* males (*d*) indicates that sperm-related regions have high ratios of genes underlying relaxed evolutionary constraint.

Despite widespread support for the “out of testis” hypothesis in insects and mammals, it is still not entirely clear to what extent novel gene formation is driven by sexual selection or by an overall permissive chromatin state. A comprehensive, phylotranscriptomic analysis in mammals supported the idea that the observed pattern is largely a product of the permissive chromatin state resulting in pervasive transcription (Soumillon et al. 2013). In agreement with this, we found that not only are more novel genes expressed in sperm-related regions, but that sperm-related regions also tend to express the overall highest numbers of genes irrespective of the gene’s age (supplementary fig. S3, Supplementary Material online). This observation suggests that the expression of novel genes in nematodes is also, at least partially, a byproduct of the higher transcriptomic complexity in sperm-related regions. To test if sperm-related genes in *C. elegans* are actually functional, we used signatures of purifying selection as indirect evidence of function (Prabh and Rödelsperger 2016). Comparison of nonsynonymous versus synonymous substitutions (d*N*/d*S*) of orthologous pairs between *C. elegans* and *C. briggsae* indicated that basically all *C. elegans* genes with *C. briggsae* orthologs exhibit strong purifying selection, which is indirect evidence of biological function (Prabh and Rödelsperger 2016) (fig. 6). Contrasting different phylostrata, we found that younger genes are generally less constrained than genes of more ancient phylostrata, which is consistent with previous phylogenomic analysis of *P. pacificus and Pristionchus exspectatus* orthologs (Prabh et al. 2018) (fig. 6*a*). When estimating selective constraint across the sets of regional genes, we found in all three data sets that sperm-related regions exhibit high ratios of genes under relaxed evolutionary constraint (fig. 6*b*–*d*). Thus, pervasive transcription but also rapid evolution, seem to be inherent features of sperm-related regions.

### Young *Out of Testis* Genes Affect Spermatogenesis and Meiosis

Finally, to further support the idea that recently evolved “out of testis” genes are not just a byproduct of pervasive transcription but actually got integrated into the biology of their host (Rödelsperger, Prabh, et al. 2019), we exploited the enormous genetic knowledge of *C. elegans* to screen for novel genes with well-characterized biological functions. Although the largest portion (∼90%) of novel genes (Phylostrata I–III) has not even been characterized in *C. elegans*, we found a number of recently evolved genes with functions in spermatogenesis and meiosis (table 1). Notably, we found regional expression of several meiosis-related genes such as *him-5* (Meneely et al. 2012), *lab-1* (Tzur et al. 2012), *syp-2* and *syp-3* (Schild-Prüfert et al. 2011), *kca-1* (McNally et al. 2012), *mesp-1* (Wolff et al. 2016), *brc-2* (Checchi et al. 2014), and *ify-1* (Kitagawa et al. 2002) in sperm-related regions C6 and C10 of *C. elegans* hermaphrodites. The same regions also express other spermatogenesis-related genes such as *spe-11* (L’Hernault et al. 1988) and *fog-2* (Nayak et al. 2005). In region CM7 of *C. elegans* males, we found expression of multiple novel spermatogenesis and meiosis-related genes such as *spe-12* (Nance et al. 1999), *spe-27* (Minniti et al. 1996), *spe-11* (L’Hernault and Singson 2000), *spe-19* (Geldziler et al. 2005), *spe-38* (Chatterjee 2005), *lab-1* (Tzur et al. 2012), and *ify-1* (Kitagawa et al. 2002) as well as nine members of another uncharacterized nematode-specific protein family (NSP group A, table 1). These results demonstrate that multiple seemingly newly arisen genes have been integrated into the biology of their host and modulate universal processes including spermatogenesis and meiosis.

**Table 1.** Candidate List of Novel, Sperm-Related *Caenorhabditis elegans* Genes.

Category	*C. elegans* Genes
Meiosis	*him-5*, *cdc-26*, *lab-1*, *kca-1*, *syp-2*, *syp-3*, *mesp-1*, *brc-2*, *ify-1*, *hcp-2*, and *rsa-2*
Spermatogenesis	*fog-2*, *spe-27*, *spe-19*, *spe-11*, *spe-38*, and *sss-1*
Other F-box proteins	*fbxb-38*, *fbxb-36*, *fbxc-20*, *fbxa-201*, *fbxa-196*, *fbxa-218*, *fbxa-125*, *fbxc-44*, and *fbxb-52*
Nematode-specific protein group A	*nspa-1*, *nspa-2*, *nspa-3*, *nspa-4*, *nspa-5*, *nspa-6*, *nspa-7*, *nspa-8*, and *nspa-10*
Germ granules	*pgl-1*, *pgl-2*, and *pgl-3*
Others	*sdz-27*, *hil-8*, *cec-2*, *numr-2 egl-1*, *dlc-6*, *kbp-1*, *mut-16*, *hmg-12*, *hpo-40*, *gpr-1*, *gpr-2*, *clec-260*, and *clec-142*

Note.—*Caenorhabditis elegans* genes of phylostrata I–III that have been characterized previously.

## Discussion

Nematodes are one of the most species-rich animal phyla and with *C. elegans*, they harbor one of the best studied model organisms (Lambshead 1993; Blaxter 1998). Many of the free-living nematodes, including *C. elegans*, are characterized by their small body, which was one the key features for the selection and success of *C. elegans* as a model system. However, for a long time, their small body size has also hindered the generation of large-scale tissue-specific expression catalogs that are comparable to mammals (Dupuy et al. 2007; Spencer et al. 2011; GTEx Consortium 2013; Smith et al. 2019). With the advent of spatial transcriptomics and single-cell sequencing, the nematode research community can now overcome this hindrance (Cao et al. 2017; Ebbing et al. 2018). Although single-cell RNA-seq still has obvious advantages in terms of resolution, spatial transcriptomics provides additional positional information that is very useful to disentangle signals from symmetric anatomical structures such as the hermaphroditic gonads. In this study, we combined cryosectioning of individual *P. pacificus* worms with RNA-seq in order to study the evolution of novel genes at spatial resolution and to establish a resource for comparative studies of gene function between *C. elegans* and *P. pacificus* (Markov et al. 2016; Moreno et al. 2017, 2018). Since individual sections represent a mix of heterogeneous tissues and cells (e.g., cuticle, neurons, intestine, somatic gonad, and germline), we focused on the analysis of regional genes with enriched expression in a given region relative to the whole worm in order to minimize contributions from other tissues. However, further experimental analysis would be needed to confirm the expression of these regional transcripts in the proposed tissue. By comparison with the spatial transcriptome of *C. elegans* hermaphrodites, we found that only a small number of one-to-one orthologs show conserved regional expression. This result resembles recent findings of expression divergence between the developmental transcriptomes of *C. elegans* and *C. briggsae* (Lu et al. 2020) and together, these results suggest that nematode transcriptomes harbor considerable species-specific signals at a spatial and temporal level. However, further analysis will be needed to disentangle to what extent the limited spatial conservation is caused by expression divergence, heterogeneous composition of cells and tissues across sections, lineage-specific duplications, or low expression levels that are below the current limits of detection (individual sections of our spatial transcriptome are sequenced at a substantially lower depth than samples in previous RNA-seq studies [Baskaran et al. 2015; Lightfoot et al. 2016]). At the same time, the limited level of conserved regional expression highlighted important differences between species, including the gain of spatially restricted expression of complete gene families. Focusing on sperm-related regions, we identified some previously uncharacterized gene families that might play roles in spermatogenesis or sperm cell biology (fig. 4) and we found broad support for the “out of testis” hypothesis in nematodes (fig. 5). This is particularly interesting as unlike *Drosophila* and most mammals, *C. elegans* and *P. pacificus* are androdioecious species where most individuals are hermaphrodites that are capable of self-fertilization and only a small percentage of a population is composed of males. Hermaphrodites of both species produce spermatids during larval development which are activated upon contact with oocytes that are formed after transitioning to the adult stage (L’Hernault 2006). Although these spermatids are transcriptionally and translationally quiescent (no de novo transcription and translation), transcripts are still detectable which allowed us to investigate the “out of testis” hypothesis in young adult hermaphrodites of both species. Note however, that the enrichment of novel genes in sperm-related regions was strongest in the spatial transcriptomic data of *C. elegans* males with active spermatogenesis (fig. 5*c* and supplementary fig. S2, Supplementary Material online). Interestingly, the evolution of selfing in nematodes led to genome contractions and gene losses that preferentially affect male-biased and sperm-related genes (Fierst et al. 2015; Rödelsperger et al. 2018; Yin et al. 2018). Thus, the analysis of gonochoristic sister species could reveal sperm-related expression of further novel genes that have been lost in the *P. pacificus* and *C. elegans* lineages.

Gene duplication is a major source for generating novel genes and functions (Ohno 1970), but other processes such as divergence and de novo formation can also generate novel orphan genes (Schlötterer 2015; McLysaght and Hurst 2016; Van Oss and Carvunis 2019) and first cases of these processes have been recently characterized in nematodes (Prabh and Rödelsperger 2019; Rödelsperger, Prabh, et al. 2019; Zhang et al. 2019). The spermatogenesis-related gene *fog-2* is a good example of sequence divergence. Mutations in *fog-2* transform hermaphrodites into females that are not capable of self-fertilization (Schedl and Kimble 1988). *fog-2* arose recently via gene duplication in *C. elegans* and has no one-to-one ortholog in *C. briggsae* (Nayak et al. 2005). According to our phylostratigraphic analysis, it is classified as *Caenorhabditis* specific. However, the FOG-2 protein and also other sperm-related genes (table 1) contain a widely distributed *F-box* motif that is also recognized by searches using profile hidden Markov models (El-Gebali et al. 2019), supporting the notion that the absence of sequence similarity is due to divergence. Similarly, BRC-2 has been shown to be homologous to the mammalian tumor suppressor BRCA2 (Bork et al. 1996), suggesting that the actual gene age of highly divergent members of ancient gene families can be underestimated in phylostratigraphic analysis (Moyers and Zhang 2015). However, even if more sensitive homology searches can still detect some level of sequence similarity, we would argue that such a sequence has diverged enough from its ancestor to be called novel (Rödelsperger, Prabh, et al. 2019). On top of the lack of strong evolutionary constraint, positive selection can additionally drive sequence divergence, especially in the context of reproductive genes (Swanson and Vacquier 2002; Kasimatis and Phillips 2018). Consistent with this, previous analysis has found evidence for positive selection acting on *fog-2* (Nayak et al. 2005). Interestingly, *fog-2* is not the only F-box gene and many of its paralogs are also expressed in sperm-related regions (table 1). Thus, following the argument that spatial expression is indicative for biological function (Ebbing et al. 2018), we would also hypothesize that these F-box genes might play some previously undescribed roles during spermatogenesis. Nevertheless, not every gene that is found to be coexpressed with MSPs is likely to perform biological functions in sperm cells. The high transcriptional complexity in sperm-related regions as a result of a permissive chromatin state may lead to the expression of putative de novo genes without any specific function. Thus, the transcriptome of sperm-related regions might harbor a mix of many classes of novel genes some of which are simply byproducts of pervasive transcription. Even if such sequences do not have any biological function, they form the raw material out of which new genes may evolve. Some of these potential de novo genes may start to interact with other cellular components and gain biological functions (Keeling et al. 2019). At the same time, recent gene duplicates and rapidly evolving members of ancient gene families also contribute to this heterogenous pool of novel genes that is highly expressed in sperm-related regions. This is further supported by the recent finding in mammals that even though error correction processes such as transcriptional scanning exist, male reproductive genes exhibit an unusually high level of sequence divergence (Xia et al. 2020). In future, more detailed analysis and functional studies of individual genes and gene families are needed to comprehensively characterize the roles of novel genes in sperm cell biology.

## Conclusion

By comparison of the spatial transcriptomes between *P. pacificus* and *C. elegans*, we have shed light on the evolution of novel genes in a tissue-specific context. We present highly spatially restricted expression profiles for many previously uncharacterized gene families in *C. elegans* and *P. pacificus*, which might help future work to study their function in a more targeted context. Focusing on sperm-related genes, we provide first evidence in nematodes supporting the previously postulated “out of testis” hypothesis and demonstrate that the sequences expressed during spermatogenesis represent a mix of biologically relevant genes as well as potential byproducts of pervasive transcription. Furthermore, we find evidence for rapid evolution of sperm-related genes at the level of sequence and copy number, which reflects typical patterns of the evolution of reproductive genes. Exploiting the wealth of genetic knowledge in *C. elegans*, we could show that even universal cellular processes such as meiosis are affected by the birth of novel genes. Finally, our data set may be taken as a starting point to investigate how gene expression changes and genomic novelty coincide with morphological and behavioral diversity.

## Materials and Methods

### Worm Culturing and Sample Preparation

*Pristionchus pacificus* strain PS312 was grown on OP50 *Escherichia coli* strain on S-medium-agar petri plates (Stiernagle 2006; Werner et al. 2017). Bleached egg populations were seeded on to plates to obtain synchronized worm cultures. Worms were screened ∼64 h post bleaching, to determine their developmental stage, under a high-magnification stereomicroscope. Young adults were sought out for, by looking out for the invaginated vulval shape and absence of embryos in the germline (Sommer 1997; Mok et al. 2015), and were selectively picked from the plates for subsequent cryosectioning and sample preparation steps.

### Cryosectioning and Preparation of Sequencing Libraries

Young adult worms were straightened in cryosolution (OCT). The head and the tail were marked by a single blue bead (Affigel Blue Gel agarose beads). The worms were then frozen, within 10 min of preparation, on dry ice and stored at −80 °C prior to cryosectioning. The embedded worms were sectioned using a cooled histology cryotome (−20 °C). Sections of 20 μm width were made starting from the first blue bead until the last blue bead. Typically, a worm, in-between two blue beads, would comprise about 40 sections. The sections of one individual worm were placed in a cooled pre-prepared 96-well plate, previously stored at −80 °C. The 96-well plates contain barcoded primers used for the CELseq protocol; consisting of a PolyT stretch, a unique 8-nt section-specific barcode, a 4-nt random barcode, the 5′ Illumina adaptor, and a T7 promotor for in vitro transcription. 96-Well plates containing sectioned worms were sealed, spinned down at 300 rpm at 4 °C, and stored at −80 °C. mRNA processing, reverse transcription, in vitro transcription, and illumina sequencing library preparation were performed according to the robotized version of the CELseq2 protocol (Hashimshony et al. 2012, 2016; Muraro et al. 2016), by the Single Cell Facility at the Hubrecht Institute. Sample processing was performed within a week of cryosectioning.

### Preparation of a Reference Transcriptome

As current gene annotations for *P. pacificus* do not include 3′UTRs (Rödelsperger, Athanasouli, et al. 2019), we generated a customized *P. pacificus* reference annotation based on a previously established strand-specific transcriptome assembly (Rödelsperger et al. 2016, 2018). To reduce isoform redundancy, we applied the following set of rules to pick one representative isoform per gene: 1) pick the isoform that had a poly-A tail which was defined as the last six nucleotides or more being adenine, 2) in case of multiple isoforms with a poly-A tail, the isoform that had the most 3′ alignment to the best protein hit in *C. elegans* was chosen, 3) if neither rule 1 nor rule 2 could resolve the ambiguity, we picked the longest isoform. Open reading frames were then extracted from these sequences by taking the longest partial or complete translatable peptide sequence that was not interfered by a stop codon (Rödelsperger et al. 2018). We then applied an Inparanoid-like approach of detection of one-to-one orthologs with *C. elegans* (Remm et al. 2001). In summary, BlastP searches against combined protein data from both species were performed (*e*-value <0.001) and only best-reciprocal pairs with lowest cross-species *e*-value (self-hits were excluded) were reported to avoid calling one-to-one orthologs in the presence of inparalogs. Finally, to annotate protein domains in the reference annotation, we ran the hmmsearch program of the HMMER3.0 package (-E 0.001) (Mistry et al. 2013).

### Analysis of Tomo-Seq Data Expression Data

Raw reads were demultiplexed, aligned to the reference annotation, and transcript counts were quantified as described in Ebbing et al. (2018). Transcript counts were normalized by the total number of transcripts per section. Subsequently, for each gene in a single worm, *z*-scores were calculated using the scale function as implemented in R on the transcript normalized expression values across all sections. To make sure that the *P. pacificus* and *C. elegans* data sets are maximally comparable, we reanalyzed the previously generated spatial transcriptomics data of *C. elegans* males and hermaphrodites (Ebbing et al. 2018) using the same normalization procedures and rules for defining the anatomical regions. We then used the expression pattern of selected marker genes to classify the sections into regions. Data from multiple individuals were combined by pooling all sections corresponding to a certain region and taking the median *z*-score from all these sections. Clustering of sections based on Pearson correlation and clustering of regions based on *z*-scores of one-to-one orthologous genes were done using complete linkage clustering as implemented in the hclust and heatmap functions of R. The tomo-seq data of *C. elegans* hermaphrodites and males were analyzed in the same way. Similarly, spatial transcriptomic data from the study of Tzur et al. (2018) were extracted from supplementary tables S2 and S10, Supplementary Material online, and were *z*-score normalized. For all data sets, expressed genes were defined as genes with a positive median normalized transcript count of all sections corresponding to a region and regional genes were defined as genes with a *z*-score >1. Overrepresentation analysis of protein domains (results of hmmsearch) was done by calculating the intersection between a set of regional genes and a given protein domain and quantifying the significance by performing a Fisher’s exact test against a background of all expressed genes. To correct for multiple comparisons, *P* values were adjusted by the false discovery rate method.

### Phylostratigraphic, Phylogenetic, and Selection Analysis

Phylostratigraphic analysis for *C. elegans* was performed by obtaining annotated protein sets for *C. elegans* (WS260), *C. briggsae* (WS260), *C. monodelphis* (JU1667 v1), *Diploscapter coronatus* (WBPS10), *Ancylostoma ceylanicum* (WS248), *P. pacificus* (WS260), *Bursaphelenchelus xylophilus* (WS248), *Ascaris suum* (WS248), and *Trichinella spiralis* (WS248). The set of *C. elegans* proteins (longest isoform per gene) were taken as query sequences and phylostrata were defined from the pairwise BlastP (version 2.6) searches against all other species (*e*-value <0.001) (Domazet-Loso et al. 2007). Phylostratigraphy for *P. pacificus* was done analogously using the predicted proteins from recently sequenced diplogastrid genomes (Prabh et al. 2018) and proteins from *C. elegans*, *Bursaphelenchelus xylophilus*, *Ascaris suum*, and *Trichinella spiralis* as target BlastP databases (*e*-value <0.001). To account for potentially incorrectly annotated open reading frames (ORFs) in the *P. pacificus* reference annotation, additional BlastN searches against the corresponding diplogastrid transcriptome assemblies (Rödelsperger et al. 2018) were performed (*e*-value <10^−5^). Phylogenetic analysis of selected gene families was done by extracting *C. elegans* and *P. pacificus* protein sequences based on the protein domain annotation created by hmmsearch. Further sequences were included after manual Blast searches on pristionchus.org, wormbase.org, and parasite.wormbase.org. A multiple sequence alignment was then generated by the MUSCLE program (version 3.8.31) and maximum likelihood trees were reconstructed using the phangorn R package (pml and optim.pml function with the following parameters, model = “LG,” optNni = TRUE, optBf = TRUE, and optInv = TRUE). One hundred bootstrap pseudoreplicates were calculated and edges with support values above 90 were labeled in the resulting trees using the Dendroscope and Inkscape softwares. In order to quantify the strength of selection, we first computed orthologous clusters between *C. elegans* and *C. briggsae* proteins, *P. pacificus* and *P. exspectatus* proteins, and *P. pacificus* and *Pristionchus fissidentatus* proteins with the help of orthAgogue (Ekseth et al. 2014). Subsequently, pairs of orthologous proteins were aligned by MUSCLE (version 3.8.31) (Edgar 2004) and translated into a codon alignment by the program pal2nal (version 14) (Suyama et al. 2006). We then ran the codeml program of the paml package (version 4) to estimate one d*N*/d*S* value for each orthologous pair (codeml model 0) (Yang 2007). For each query gene for *C. elegans* and *P. pacificus*, we selected the ortholog with lowest estimated d*S* as one-to-one ortholog for further selection analysis. d*N*/d*S* values for subsets of genes, for example, different phylostrata or sperm-related genes, were then visualized as boxplots in R. For better comparison of d*N*/d*S* values across anatomical regions and nematode genera, we chose a pair of *Pristionchus* species that is separated by similar evolutionary distance (measured as median d*S*) as *C. elegans* and *C. briggsae.* As median d*S* between *C. elegans* and *C. briggsae* was 2.1, the matching *Pristionchus* species pair corresponded to *P. pacificus* and *P. fissidentatus* (median d*S* = 1.9) which was considerably more diverged than *P. pacificus* and *P. exspectatus* (median d*S* = 0.3).

## Supplementary Material

Supplementary data are available at *Molecular Biology and Evolution* online.

## Supplementary Material

msaa207_supplementary_data

## Notes

Sequencing data have been submitted to the European Nucleotide Archive under the study accession PRJEB37439.
