# Supplementary material for: Spatial Transcriptomics of Nematodes Identifies Sperm Cells as a Source of Genomic Novelty and Rapid Evolution
Source: Mol Biol Evol. 2020 Aug 8;38(1):229–43. doi: 10.1093/molbev/msaa207 (PMC8480184; doi:10.1093/molbev/msaa207)
Supplement: msaa207_supplementary_data [file msaa207_supplementary_data.zip › Supplementary_Figures.pdf]

Supplementary Material for

Spatial transcriptomics of nematodes identifies sperm cells as a source of genomic novelty and rapid evolution

Christian Rödelsperger[1,5,*], Annabel Ebbing[2,5], Devansh Raj Sharma[1], Misako Okumura[4], Ralf J. Sommer[1], Hendrik C. Korswagen[2,3,*]

[1] Department for Integrative Evolutionary Biology, Max Planck Institute for Developmental Biology, Max-Planck-Ring 9, 72076 Tübingen, Germany, [2] Hubrecht Institute, Royal Netherlands Academy of Arts and Sciences and University Medical Center Utrecht, Uppsalalaan 8, 3584 CT Utrecht, The Netherlands, [3] Institute of Biodynamics and Biocomplexity, Developmental Biology, Department of Biology, Utrecht University, Padualaan 8, 3584 CH, Utrecht, The Netherlands, [4] Program of Biomedical Science, Graduate School of Integrated Sciences for Life, Hiroshima University, 1-3-1 Kagamiyama, Higashi-Hiroshima, Hiroshima, 739-8526, Japan,
[5] these authors contributed equally, [*] Corresponding authors' email addresses: christian.roedelsperger@tuebingen.mpg.de and r.korswagen@hubrecht.eu

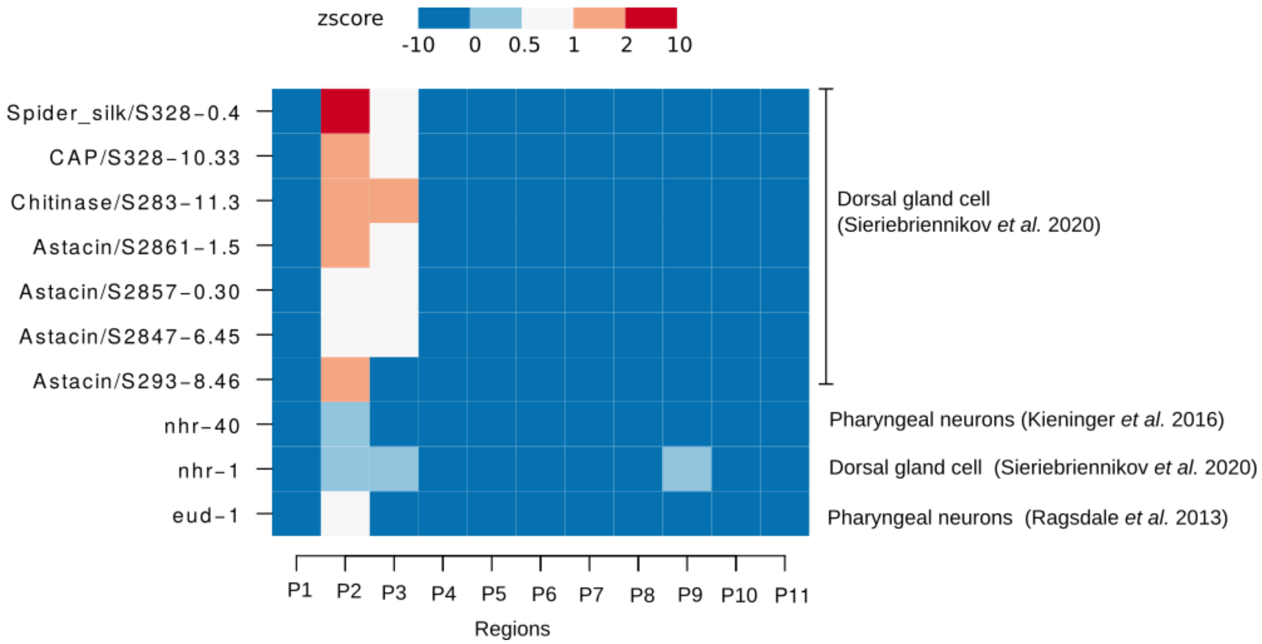

Fig. S1 Comparison of spatial transcriptome data with reporter lines of ten pharyngeal genes in *P. pacificus.* For all ten genes, expression is detected in regions P2 and P3.

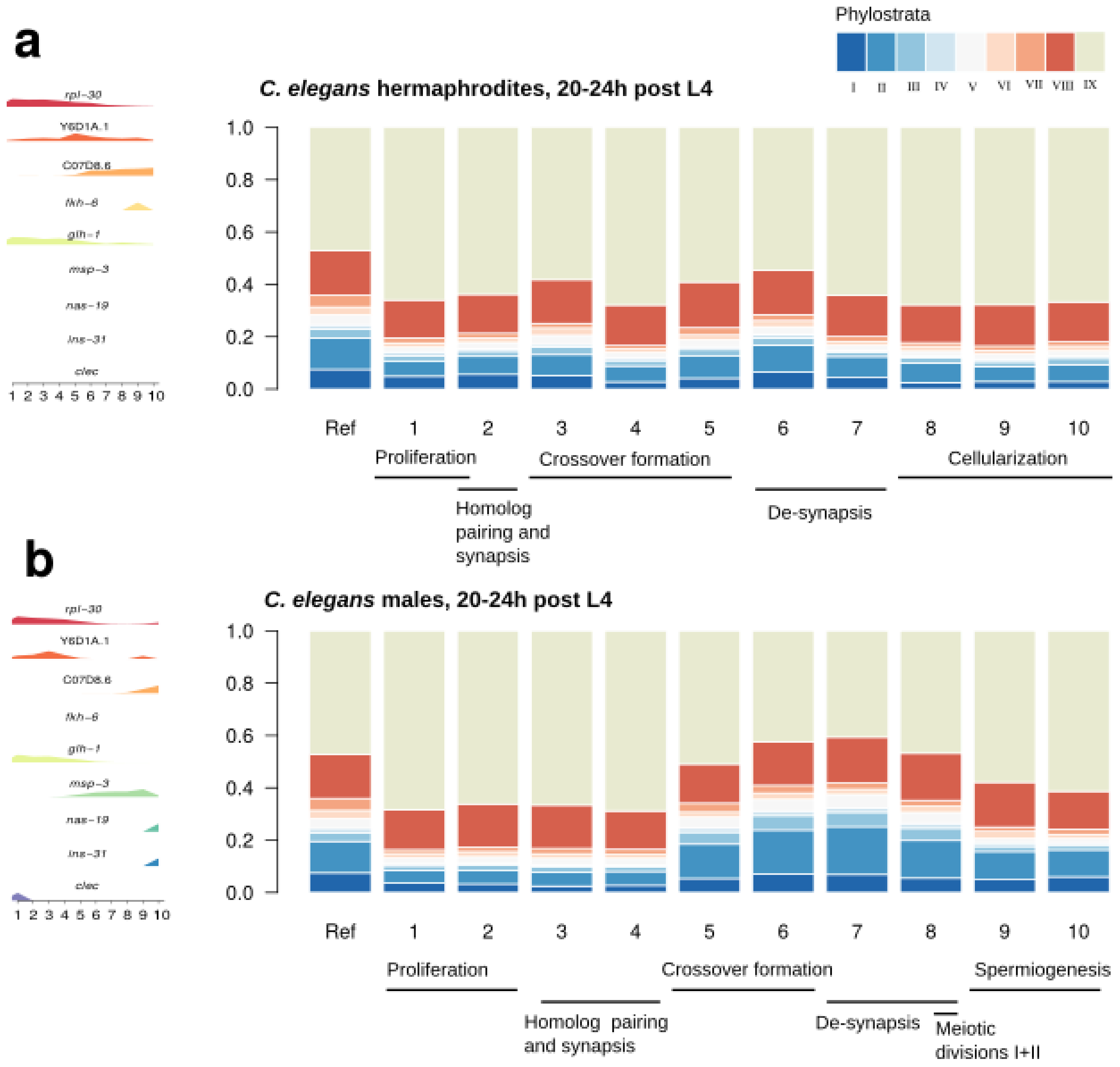

Fig. S2 Phylostratigraphic analysis of regional genes in spatial transcriptomes of tens laser-dissected germline sections. **a** Expression of selected marker genes and phylostratigraphic analysis of *C. elegans* hermaphrodite data from Tzur *et al.* (2018). **b** Expression of selected marker genes and phylostratigraphic analysis of *C. elegans* male data. Annotations of meiotic processes are taken from Tzur *et al.* (2018).

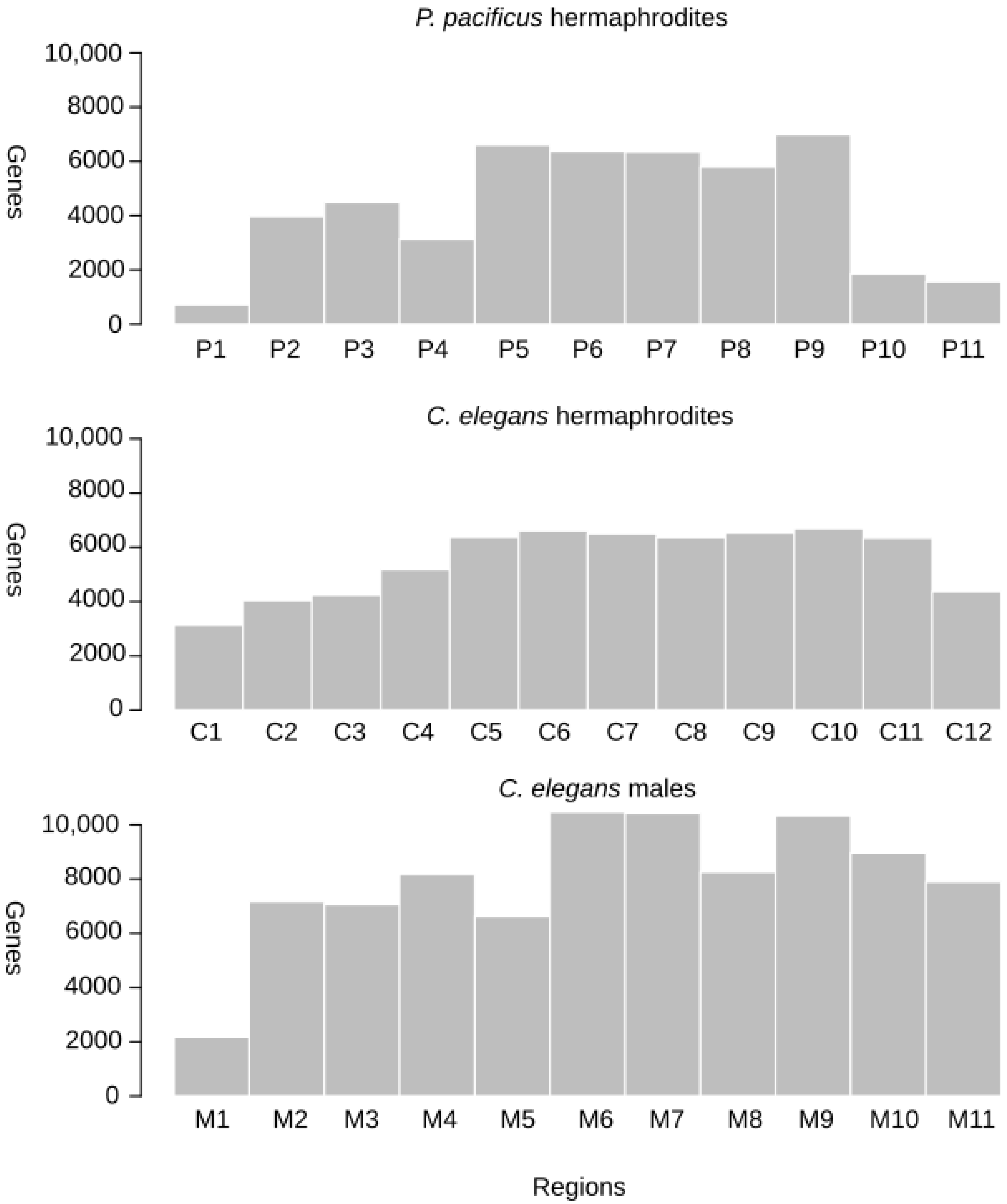

Fig. S3 Pervasive transcription during spermatogenesis. The high numbers of expressed genes in sperm-related regions (P5, P9, C6, C10, M7) suggests that expression of certain novel genes may be a byproduct of pervasive transcription.
